# Goldsurfer2 (Gs2): A comprehensive tool for the analysis and visualization of genome wide association studies

**DOI:** 10.1186/1471-2105-9-138

**Published:** 2008-03-04

**Authors:** Fredrik Pettersson, Andrew P Morris, Michael R Barnes, Lon R Cardon

**Affiliations:** 1 Dept Bioinformatics, Wellcome Trust Centre, Oxford, UK; 2 Molecular Discovery Informatics, GlaxoSmithKline Pharmaceuticals, Harlow, Essex, UK; 3 Fred Hutchinson Cancer Research Center, Seattle, Washington, USA

## Abstract

**Background:**

Genome wide association (GWA) studies are now being widely undertaken aiming to find the link between genetic variations and common diseases. Ideally, a well-powered GWA study will involve the measurement of hundreds of thousands of single nucleotide polymorphisms (SNPs) in thousands of individuals. The sheer volume of data generated by these experiments creates very high analytical demands. There are a number of important steps during the analysis of such data, many of which may present severe bottlenecks. The data need to be imported and reviewed to perform initial quality control (QC) before proceeding to association testing. Evaluation of results may involve further statistical analysis, such as permutation testing, or further QC of associated markers, for example, reviewing raw genotyping intensities. Finally significant associations need to be prioritised using functional and biological interpretation methods, browsing available biological annotation, pathway information and patterns of linkage disequilibrium (LD).

**Results:**

We have developed an interactive and user-friendly graphical application to be used in all steps in GWA projects from initial data QC and analysis to biological evaluation and validation of results. The program is implemented in Java and can be used on all platforms.

**Conclusion:**

Very large data sets (e.g. 500 k markers and 5000 samples) can be quality assessed, rapidly analysed and integrated with genomic sequence information. Candidate SNPs can be selected and functionally evaluated.

## Background

With recent advances in the efficiency of high-throughput SNP genotyping technology, genome-wide association studies are now routinely undertaken with hundreds of thousands of SNPs genotyped on sample sizes necessary to detect the modest genetic effects we expect for complex diseases [1-4]. There is now a clear demand for efficient tools that allow processing of the data generated by these studies, including QC, statistical analysis and subsequent evaluation, visualization and interpretation of results. To meet these demands, we have developed Goldsurfer2 (Gs2), a new integrated software package for GWA analysis, developed from the Goldsurfer tool [5]. The main feature of the original version of the Goldsurfer program was its 3D visualisation of LD patterns. While this functionality is still available and has been further developed in Gs2, the focus of program has been shifted towards performing and evaluating genetic association testing. The main improvements from the previous version of Goldsurfer are the ability to work on a genomewide scale, performance and feature wise, and added methods for statistical analysis and visualization of results.

The two single most important factors leading to the current surge in GWA studies are the advances in chip-based genotyping technologies [6,7] and the available data from the HapMap project, in which a large number of common genetic variations were characterized and genotyped for a panel of four different human population samples [8]. The application of chip-based technology allows cheap, quick and readily available measurement of a large subset of the SNPs characterised by the HapMap. Standard analysis is now being routinely undertaken using up to 650000 markers and is even cheaper than customized analysis of a smaller number of markers. A chip containing 1 million SNPs is now under development and likely to be available soon. Although chip-based technology has been used for quite some time for measuring gene expression, measurement of genotypes generates different analytical challenges. Studies of gene expression normally measure a relatively low number of samples, however GWA studies can easily involve many thousands of samples to provide sufficient statistical power of the analysis. This leads to new analytical challenges, as the dimensionality of data sets increase.

Another distinguishing feature of SNP analysis compared to gene expression analysis is that mRNA transcripts have mostly been experimentally verified to be expressed in various tissues and many will have a known biological role. By contrast, most SNPs have no defined functional role and can be located in coding as well as non-coding regions of the genome. To make biological sense of the findings from GWA studies it is crucial to link results to available biological annotation, for example by comparing the location of SNPs relative to genes and biological features such as CpG islands and recombination hotspots. It may be particularly important to dissect the functional role of a SNP in the full context of the surrounding genomic sequence, for example if it is found in or near a gene, in an intron, exon or regulatory element. Further analysis of candidate genes may involve looking at regulatory pathways, studying expression profiles, and the biological role, cellular location and molecular function based on annotation using the controlled vocabulary of gene ontology (GO) [9].

Before defining useful candidate markers and genes a GWA project will involve many nontrivial steps. To optimise a study in terms of power and to avoid confounding factors it is crucial to have a well-designed experiment with a large enough sample size number and a well-characterised phenotype. With the new large-scale high throughput technologies the problem has moved from generating the data itself to actually making sense of the wealth of information hidden behind a background of type I error caused by multiple testing. With the large datasets it is even difficult to store and to get an overview of the data since it is impractical to use simple text editors and that the dimensions of datasets exceed the maximum limits of data manipulation tools like Microsoft Excel. Specialised software solutions and/or database systems needs to be used to analyse the data, which demand specific data analytical and computing skills from researchers.

The statistical methods currently applied in association studies are highly sensitive to poorly conditioned data and can easily give spurious associations so it is important to perform initial quality control. Problems with the quality of the data can also be caused by the genotyping technology, including difficulties in the calling of genotypes from the raw intensities. The detection of real genetic effects can also be confounded when a marker does not conform to expectations of Mendelian inheritance, for example in the case of copy number variation. Most GWA studies are typically enriched with common genotypes as they are generally based on the markers identified by the HapMap project, a survey of common variation [8].

The variables normally used in quality control include the failure rate of genotyping for markers and samples, minor allele frequencies, differential call rates for cases and controls, skewed heterozygosity distributions and deviation from Hardy Weinberg equilibrium [10]. SNP genotype data can be said to be pseudo-binary involving a step where the original intensities are analysed using clustering algorithms. Unfortunately the calling of the genotypes using different clustering algorithms are far from perfect which means that, for example, obviously monomorphic SNPs can be erroneously assessed to have multiple genotypes, while potentially interesting candidates may be wrongly excluded as missing values.

Various methods are used for obtaining a statistical measurement of the association between a SNP represented by alleles or genotypes and a phenotype of which most are based on simple chi^2 ^statistics from genotype-by-disease tables, using tests such as the Cochran Armitage trend test. Examples of other popular methods include Fisher's exact test and logistic regression [11]. Hits are often ranked by their statistical significance in terms of Bayes factors or p-values. Although association with SNPs with low p-values are theoretically less likely to be observed by chance, when 500 K markers are tested, we can expect a very large number of preliminary associations, the vast majority of which are likely to be false. High density GWA analysis accentuates the need for new approaches to follow up preliminary results to find candidates that are more likely to be true positives. One common way of raising the significance threshold is to apply Bonferrroni correction but this may be overly conservative and could lead to discarding real hits. Another approach to obtain significance limits is to perform permutation testing which is less conservative but more computationally demanding.

Genetically associated markers often show a high degree of correlation with each other. Neighbouring markers often do not vary independently from each other, which are referred to as linkage disequilibrium (LD). Analysing LD patterns can be useful for disentangling the underlying mechanism of an association. A suggested significant marker is not necessarily the causative variant but can be in LD with an ungenotyped functional variant. By comparing findings to the LD structure in the HapMap, studies can easily be expanded to find SNPs outside the genotyped subset. Another interesting way of interpreting LD is to compare patterns between different populations or different classes of samples such as cases and controls. Looking at LD patterns and comparing with genomic annotation features in candidate regions can be interesting for unravelling the functional explanation of a potential finding.

As previously mentioned there is a great need for specialised software for GWA studies. Most available software for performing statistical analysis of genotype-phenotype datasets are mainly command line based and do not tend to be very user friendly for inexperienced computer users although the area is improving with some recent products, Haploview [12] and Plink [13]. Also the scale of the data makes its representation as plots and tables far from straightforward or even impossible. Another problem with currently available tools is that a large number of different tools are needed to perform genotype QC, statistical analysis and downstream interpretation. Genomizer [14] is another tool that is similar to Gs2 in that it is implemented in Java and that it can be used for data import, statistical analysis and evaluation of results. It focuses on importing genotyping calls from Affymetrix GeneChip arrays and employs a different approach to plotting the results by opening generated image files in an external browser.

One of the main workloads for analysis in statistical genetics and bioinformatics is the frequent formatting of data for export and import of very large data files. This procedure involves writing and running new programs and scripts that are often time consuming in both implementation and execution, creating numerous opportunities for the introduction of errors due to the data manipulation. In general there is a need for a single efficient and user-friendly platform that can accommodate multiple steps of the data analysis process from data management to evaluation of results using biologically relevant information and interactive plotting facilities.

## Implementation

Gs2 is implemented as a platform independent Java 1.5 application that can be used locally on the machine on which it is installed. The program is run by downloading the application or by using Java Start. No additional libraries besides those that come with the installation need to be downloaded. To link to and retrieve genome annotation information from external sources such as the UCSC database [15] users need to provide path and connection information to a suitable mirror of it. The installation also comes with precompiled flatfiles containing basic biological annotation and more updated and complete versions of these will be available to download from the project webpage.

**Figure 1:**
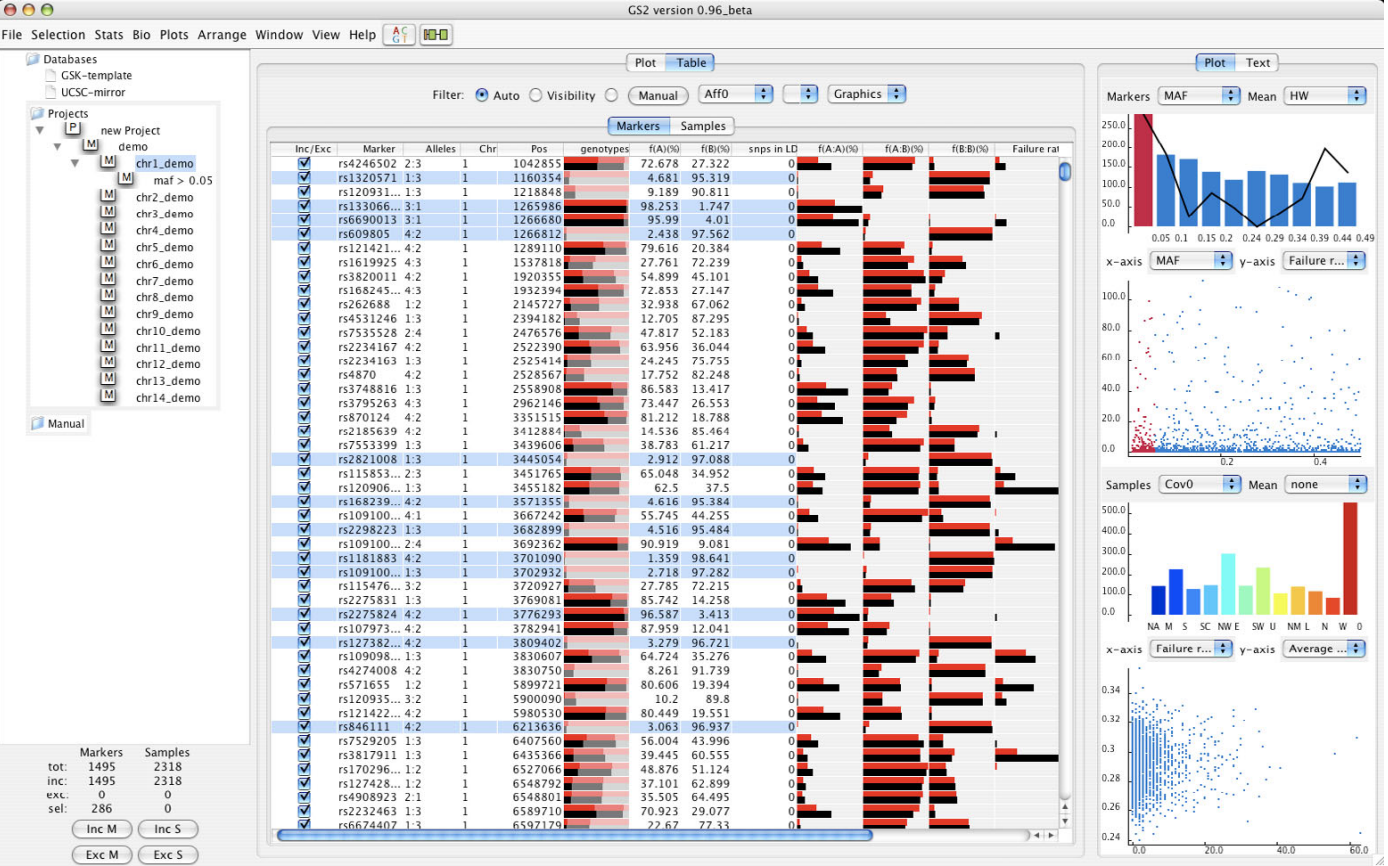
The graphical user interface of Gs2 makes it easy to analyse whole projects by arranging data in a hierarchical structure and by providing interactive plots and tables for summarising data. In this example data have been loaded by importing multiple genotype files, one for each chromosome. For the selected node, rare markers with minor allele frequency below 5% have been selected and a new cleaned dataset has been created in the tree. The table and the plots show the information for the selected dataset. The bars in the table show calculated values for cases and controls respectively. The plots on the right side of the window shows, in the order of vertical appearance with the first two showing marker information and the second two sample information, distribution of minor allele frequency with average Hardy Weinberg exact test probabilities, minor allele frequency plotted against failure rate, covariate distribution by regional origin and finally failure rate plotted against sample heterozygosity.

The design of Gs2 is based around an interactive graphical user interface (Figure 1). A central part of the design is the arrangement of data in a project based tree structure. This allows for organising data by splitting up it into different categories such as the chromosomal origin, ethnic origin or affection status while still obtaining an overview of the dataset. The progress of the data analysis can easily be saved by storing subsets of data based on performed selections and exclusions/inclusions of SNPs and samples after different steps of quality control. In projects with complex data analysis processes involving many collaborating researchers it is a big risk that the analysis gets out of control due to problems with version control. We have built in functionality to keep track of the data analysis flow by automatically creating and saving timestamps when performing actions such as saving projects and formatting data. The implementation of Gs2 is optimised for analysis of very large datasets with thousands of samples and more than 500,000 markers. Following import of tab delimited files in standard or custom formats, Gs2 internally creates marker objects and sample objects in which imported and calculated values such as properties and statistics are stored in local data structures. Genotype data is written to temporary swap files that are accessed when performing subsequent calculations and are internally purged to free memory after these have been performed. For speeding up this procedure tasks are multithreaded which means that on a multi core system all processors are used for calculations. Gs2 is able to compress gigabyte-sized text data sets into megabyte-sized binary files by saving genotypes into low-level binary data format and to save this together with calculated and imported values into project files.

A range of standard methods for quality control of data and association testing have been implemented but new features such as additional statistical methods can easily be added to Gs2 through its dynamic design. There is a steady stream of novel methods developed in both industry and academia for statistical analysis of genetic data. The most long-lived and useful software will be the ones that are designed to easily accommodate new methods. Of central importance for the GUI are the interactive plots that can be used for showing all information about markers and samples and for performing selections for further analysis.

### Program Overview

In order to exemplify the features of Gs2 we will review the typical steps that may be taken during the analysis of a whole genome association project.

#### Importing data

Genotypes can for example be imported from flatfiles in the standard ped format [16] and binary bed format [13]. HapMap genotype files can also be downloaded from the official HapMap webpage [17] and imported into Gs2. Compressed genotypes together with associated calculated and imported information can also be loaded from a previously saved Gs2 project. As mentioned there are a wealth of formats available and it will be impossible to cover all specifications. For flexible import of data to Gs2 a preview function allows the user to specify how data is formatted and how it should be imported, for example to set if samples or markers are represented in rows or columns respectively and to identify columns with for example affection status. Import and analysis of multi-allelic markers and micro satellites is currently not supported. Built in, there is functionality for importing genotyping calls exported from the Affymetrix platform and importing data from other main genotyping platforms can easily be added due to flexible implementation of Gs2. Additional formats not currently supported have to be transformed into one of the standard formats that are currently supported by Gs2, ie traditional "ped" format and the recent binary ped or "bed" format developed and popularized by plink. Results from GWA studies in the form of plink output files or in publicly available summary files such as those of the Wellcome Trust Case Control Consortium project (WTCCC) [18] can be imported into Gs2 without loading genotyping calls.

Loaded datasets are organized in a hierarchical tree structure with each dataset populating the tree as an individual sub node with associated plots and tables visible in the main window (Figure 1). After importing the genotypes additional information for markers and samples can easily be added to the project. Examples of added sample information are quantitative data, binary affection status and covariates. For the markers, examples include importing statistical data that has been calculated using external server based programs or importing precompiled annotation data. All imported and calculated data can be visualised, used for interactive selections and used as arguments for statistical methods.

##### Summary

• Store and arrange projects in hierarchical tree structure

• Import projects saved in compressed .gs2 format

• Import genotype data from different file formats

◦ Ped, Bed or HapMap

◦ Preview function lets user specify import-format by specifying datatypes in columns and rows

• Import statistical results from GWA studies

◦ Plink and flatfile formats for publicly available output files

• By not storing data in memory, very large datasets can be imported and analysed

• Import additional information for both markers and samples

#### Performing calculations

Gs2 has a flexible and customizable design facilitating the expansion of functionality by adding new statistical methods to it. A number of methods for calculating statistics for SNPs have already been implemented in Gs2. The basic methods include calculating allele and genotype frequencies, minor allele frequency (maf), heterozygozity frequencies, failure rate and the Hardy-Weinberg exact test (hwe) [19]. These calculations are automatically performed for all samples and subsets of samples such as cases and controls. Another category of methods uses the affection status of samples for calculating tests for genetic association. The currently implemented method in this category is the Cochran Armitage trend test. For quantitative trait analysis standard linear regression has been implemented. A series of transformations have also been implemented, in which p-values, inverse chi^2 ^and genomic control [20] can be compared. All calculations can be done for multiple affection status categories. The rationale behind splitting up the calculations into different categories is to make it easy to combine different methods to get specific solutions. As an example, a quantile quantile plot (QQ-plot) is created by calculating chi^2 ^statistics with the Cochran Armitage test, calculating the p-value for the result and subsequently calculating the inverse chi2 value for the p-value and plotting these values against each other.

To give an overview of the LD profile in a dataset, an average value for each marker can be calculated using a sliding value approach. It is also possible to find markers in a second dataset, for example imported HapMap genotypes that are in LD with a selection of markers. Data can be filtered by removing redundant markers with a pairwise LD exceeding a user specified threshold, e.g, r^2 ^= 1 to remove identical markers.

To investigate the effects of population stratification, Principal Component Analysis (PCA) [21] has been implemented. PCA models are calculated on genotypes as represented 0, 1 and 2. Plotting the clustering of samples for the first components can reveal how the different populations relate to each other, if there are outliers in the data or if samples have been misclassified.

##### Summary

• A selection of methods for quality control, association analysis and analysis of linkage disequilibrium has been implemented

◦ Allele and genotype frequencies including minor allele frequencies and marker heterozygosity.

◦ Hardy-Weinberg exact test

◦ Cohran Armitage trend test

◦ Linear regression

◦ P-value calculations

◦ Average and pairwise calculation of LD

◦ Principal component analysis for studying stratification

• Perform calculations separately for sample classes such as cases and controls

• A flexible design makes it easy to add new methods

• Calculations are multithreaded to make use of all processors in multi core systems

#### Using plots and tables

For each dataset all imported and calculated data are summarised in a central interactive table and selection of plots (Figure 1). Markers and samples can easily be selected from any of these. The table can be used for showing values for cases and controls, respectively representing data by coloured bars giving both an overview and making it easy to spot differences between different samples categories. For example, it is easy to observe variations in allele and genotype frequencies between cases and controls. Plots include general 2D scatter plots, histograms with the option to show mean values for another variable for each of the bins.

**Figure 2:**
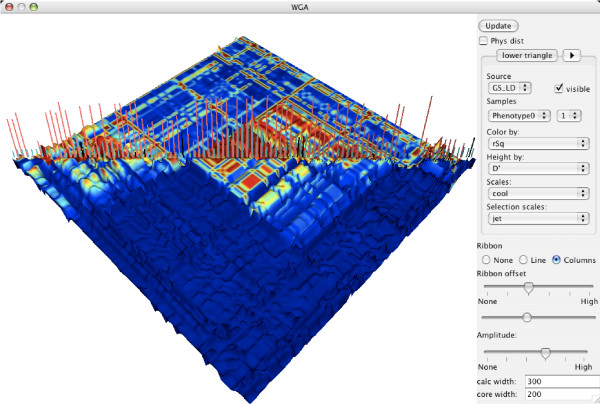
Updated GOLDsurfer 3D plot for visualising LD. The settings for rendering the upper and lower triangles of the 3D contour can be individually adjusted using the panel on the right side of the plot. In the upper triangle pairwise LD is plotted for cases only with the colour representing LD as measured by D' and the height showing r^2^. In the lower triangle LD is plotted for controls only, with r^2 ^as colour and D' as height. The column plot in the diagonal is showing, failure rate, average LD and association p-values.

**Figure 3:**
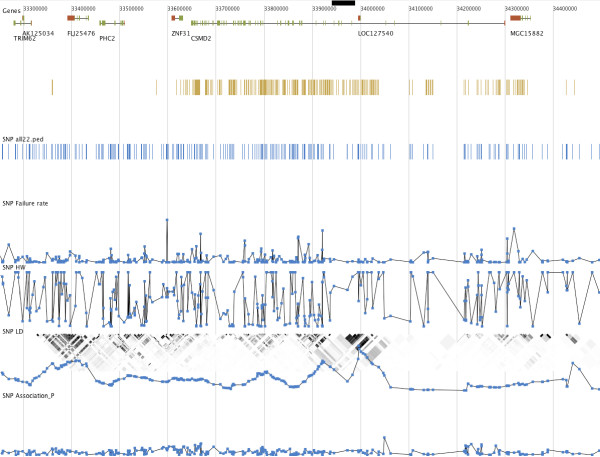
In the genome view, biological features, SNPs in dataset or in LD with selected SNPS, calculated or imported data and 2D visualisation of LD are plotted by physical position. The plot can easily be zoomed and panned both vertically and horizontally. Features and SNPs can be interactively selected by using the mouse.

**Figure 4:**
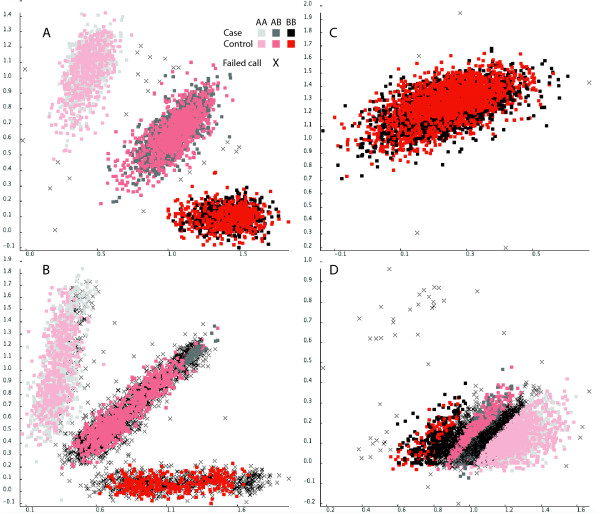
Misclassification of genotypes by clustering methods. The black and red colour scales show the genotype calls for cases and controls respectively. Black crosses represents genotypes that the clustering algorithm has called as missing values. a) Correctly called polymorphic marker b) Differential calling success between cases and control. c) A monophorphic marker with successfully assigned genotypes. d) A monomorphic marker wrongly called as being polymorphic.

Among the specialised plots is a 3D view of LD patterns (Figure 2), sample relationship by identity by state and a genome view (Figure 3) for showing marker values and annotation information as tracks such as genes, recombination hotspots and CpG islands imported from the UCSC database or local flatfile. The latter plot can be extensively zoomed both vertically and horizontally. Further information about genes and SNPs can be obtained by automatically linking to information available on the Internet. By plotting inverse against original chi^2^values from the Cochran Armitage trend test a QQ plot can easily be created to identify SNPs with unusually high association values. The 3D LD plot has been further developed from the original GOLDsurfer publication. With this plot the contour and colouring of the plot can be used to represent different measurement of LD and different parts of the plot can show calculated values for cases and controls respectively. In the diagonal calculated variables such as association p-values or maf and annotation categories can be visualised as columns or ribbons. It is also possible to plot the original intensities on which the genotyping calls are based (Figure 4). Genotyping data is typically read in traditional discrete representation and the intensities for the raw genotype type calls have to be separately imported. Plotting the intensities addresses the difficulty that a significant number of markers could have been wrongfully removed due to being called as missing values. This plot can similarly to the other plots be used to show values from for example cases and controls separately. A good approach for final quality control is to manually go through a list of the highest ranked candidates for association.

All plots can be saved as images in most common format such as tiff, jpg and png.

##### Summary

• Interactive plots

◦ 2D scatter plots

◦ Histograms

◦ Genome view

▪ View biological features such as genes with introns and exons

▪ 2D view of LD

▪ Line plots for calculated variables

◦ 3D view of LD

◦ Plot intensities for raw genotype calls

▪ Use different color schemes for different sample classes such as cases and control

• Interactive tables

◦ For easy overview of data, variables and genotype frequencies can be represented as bars in the table.

◦ Multiple sorting of markers and samples

#### Removing data failing QC

Manipulating data is a two-step process involving first selecting objects in any of the interactive plots or tables or by using the filtering tool. With the filtering tool markers and samples can be selected randomly, by entering a list of regular expressions or by setting limits for values or selecting specific classes. After the selection either inclusion or exclusion of data is performed to obtain a cleaned up version of the data.

##### Summary

• Select markers or samples using interactive plots, tables or by setting selection intervals for imported or calculated variables.

#### Selecting candidate markers

Markers can be selected based on any annotation information, imported values or calculated values or by using gene ontology terms (GO). Based on keywords genes can be selected by attributes such as name, molecular function, cellular location or biological role with markers close to these genes automatically selected.

##### Summary

• Automatically select markers close to genes selected by different annotation terms such as keywords and gene ontology.

#### Manipulating data structures

The hierarchical tree structure can be used for a structured analysis process or by splitting up data into different categories by descriptions such as chromosomal origin for markers or ethnic origin for samples. After excluding markers failing QC a new dataset can be created containing only included markers or markers in biologically interesting regions. There are many functions for manipulating the structure such as cloning, splitting and merging data. This is useful in many situations for example when samples such as cases and controls or different populations are stored in different datasets and need to be combined together. Similarly the same functionality is useful when markers are stored in separate files for example by chromosome. Using the different functions for manipulating data is useful for updating datasets after additional markers or samples have been analysed and need to be added to the previous analysis. The functions are accessed from the menu bar and the actions are applied on datasets selected in the tree structure.

##### Summary

• Use the hierarchical tree structure to keep track of the progress of the data analysis process.

#### Saving project and exporting data

The progress of the current analysis process can be saved in binary format taking considerably less space on disk compared to the original text files and keeping the structure of the project intact. Another advantage is that it takes much less time to load a saved Gs2 project compared to the initial parsing of the text files. Data can also be exported in a variety of different format such as .ped, .bed, phase to be used for subsequent analysis or just for sharing your cleaned up data.

##### Summary

• Save project in binary .gs2 file format keeping project settings, imported information and genotypes.

• Export genotypes and additional data into various formats

• Use version tracking system to keep control of actions performed in the analysis process.

## Results

To give a flavour on how Gs2 can be used in some of the important steps of genome wide association studies a couple of brief examples are presented in the sections below. For more comprehensive examples please refer to the tutorials on the project webpage.

### Investigating results from publicly available studies

Publicly available results such as those from the WTCCC can be downloaded and directly imported into Gs2. Gs2 can also import results from statistical calculations performed using Plink. It can plot interactively and query imported results such as p-values. For in-depth functional investigations, one can plot marker values together with annotated gene structures and query their functional annotation to generate summarised functional reports. To extract association p-values, a list of favourite genes can be pasted into the program to find all markers located within these target genes.

### Stratification analysis using PCA

Different genotyped populations can be batch imported from separate files. Files can be merged into a new dataset consisting of a subset of markers genotyped in all populations. This allows investigation of population stratification via PCA, after which the clustering of samples on the first components can be plotted for visual inspection. If this analysis suggests strong deleterious effects of stratification, it is not recommended to perform association testing further without accounting for the stratification.

### Association testing for genotype data

A number of procedures can be conducted for basic genotyping data. Initially, the user will import genotype data using from standard formats using built in functions and then exclude markers and samples based on quality control thresholds. A new dataset can be created with all remaining objects. Association using Cochran Armitage trend test can be done internally on these data, or externally using plink with subsequent importing of results. New sub nodes in the tree structure can then be constructed by separating markers according to chromosome. It is useful at this stage to sort markers based on the lowest association p values or largest test statistics. For QC checks of the most significant results, one can then import the raw genotyping intensities for each chromosome and plot the calls for all significant markers. For those with no obvious genotyping error the LD structure can be plotted using interactive 2D and 3D plots. These patterns can be annotated by importing HapMap data, using built-in methods for finding new markers to genotype in the more densely genotyped HapMap panel. At this stage one can import gene information, query their annotation and plot their structure and position. Finally, one can export a list with the most interesting markers and save the analysis as a Gs2 project file.

## Conclusion

Gs2 can efficiently be used on a standard laptop or desktop computer to analyse the latest generated GWA datasets, containing 500000 markers or more and as many as 5000 samples.

Loading 500000 markers and 5000 samples and performing association testing calculating the p-values for the Cochran Armitage trend test takes roughly 700 MB of memory and can be done in 15–30 minutes on a standard dual core laptop.

## Availability and requirements

Project name: Goldsurfer2

Project homepage: 

Operating system: Platform Independent

Programming language: Java

Manual: comes with download and can also be separately downloaded from project homepage

## Authors' contributions

LRC and MRB initiated and coordinated the project. APM supervised the project. FP wrote all of the code, designed the interface and wrote the manuscript. All authors read and contributed to revising the manuscript for intellectual content and approved the final manuscript.
